# Severe muscle loss during radical chemoradiotherapy for non‐metastatic nasopharyngeal carcinoma predicts poor survival

**DOI:** 10.1002/cam4.2538

**Published:** 2019-09-13

**Authors:** Xiao Huang, Jie Ma, Ling Li, Xiao‐dong Zhu

**Affiliations:** ^1^ Department of Radiation Oncology Affiliated Cancer Hospital of Guangxi Medical University Nanning Guangxi China; ^2^ Department of Radiology Affiliated Cancer Hospital of Guangxi Medical University Nanning Guangxi China

**Keywords:** chemoradiotherapy, nasopharyngeal carcinoma, sarcopenia, skeletal muscle loss, survival

## Abstract

**Background:**

Skeletal muscle loss is a novel imaging biomarker that is considered to be predictive of survival outcomes and toxicity in a variety of solid tumors. This study explored to investigate whether skeletal muscle loss after chemoradiotherapy (CRT) in nasopharyngeal carcinoma (NPC) patients can predict survival.

**Methods:**

A total of 394 non‐metastatic NPC patients were enrolled. The cross‐sectional area of the third lumbar skeletal muscle based on computed tomography (CT) scan was measured and the skeletal muscle index (SMI) was calculated. A cut‐off value suitable for the Chinese population was used to define sarcopenia, and relative changes in skeletal muscle after treatment were analyzed for the confirmation of skeletal muscle tissue loss during treatment and its impact on overall survival (OS).

**Results:**

The median follow‐up was 22.7 (range, 2.5‐46.4) months. One hundred and thirty patients (33.0%) were defined sarcopenia at baseline. Two hundred and forty one patients (61.2%) had posttreatment sarcopenia. The mean SMI before and after treatment was 42.8 and 38.1 cm^2^/m^2^ (*P* < .001), and the average SMA loss was 13.1 cm^2^. While sarcopenia before or after treatment was not associated with OS, severe muscle loss after CRT was an independent predictor of survival prognosis for NPC (hazard ratio 2.79, 95% confidence interval 1.47‐5.28, *P* = .002) when adjusted for gender and cancer stage.

**Conclusions:**

During CRT, patients with NPC often experience different levels of muscle loss, and severe skeletal muscle loss may shorten OS.

## Graphical abstract

Our results suggest that severe skeletal muscle loss during treatment is an independent risk factor for poor prognosis. Post‐treatment computed tomography reassessment of skeletal muscle is essential for prognostic analysis and screening of high‐risk population.

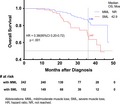

## 1 INTRODUCTION

Nasopharyngeal carcinoma (NPC) is among the most commonly observed head and neck cancers in China, with a 5‐year prevalence rate of 12.22%. In terms of morbidity and mortality, the disease ranks 18th and 17th among all malignant tumors, respectively.^1^ Radical chemoradiotherapy (CRT) is recognized as the most important treatment for non‐metastatic NPC. Side effects of CRT such as nausea, vomiting, loss of appetite, oral mucositis, and sore throat can result in reduced food intake, weight loss and even cancer cachexia. How skeletal muscle changes and its impact on treatment toxicity or survival for NPC remain unclear.

Body composition plays an increasingly important role in the field of cancer.^2^ Skeletal muscle loss is a new imaging biomarker that is increasingly being used by clinicians, especially in diseases characterized by systemic wasting, such as cancers. Despite significant advances being made in cancer research in recent years, it is still difficult to predict treatment toxicity or survival by means of routinely‐collected clinical data, such as those on age, weight loss, physical score, body mass index (BMI) and complications. Increasing evidence suggests that skeletal muscle loss is associated with poor prognoses in patients with multiple solid tumors^3^, ^4^, ^5^, ^6^, ^7^, ^8^, ^9^; however, its relationship with clinical outcomes in patients with NPC patients has not been demonstrated.

We hypothesized that skeletal muscle changes during treatment and has an impact on survival. To the best of our knowledge, this cohort study is the largest to date to explore the relationship between skeletal muscle loss and prognosis in NPC patients.

## 2 MATERIALS AND METHODS

### 2.1 Study population

**Figure 1:**
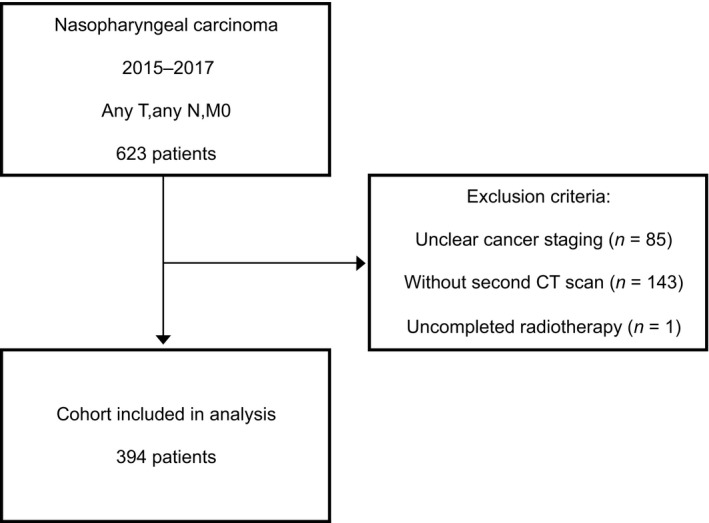
Exclusion process for admitted patients. Initially, a total of 623 patients underwent radical chemoradiotherapy. One hundred and forty three cases were excluded due to lack of second computed tomography (CT) data. Another 85 cases were excluded due to unknown staging. One patient was excluded because of incomplete radiotherapy

The research protocol was approved by the Medical Ethics Committee of the Affiliated Cancer Hospital of Guangxi Medical University, and our study was performed in accordance with the ethical standards laid down in the 1964 Declaration of Helsinki and its later amendments. We collected clinical medical record data on NPC patients who were treated at the facility between January 2015, and December 2017. The age at diagnosis ranged from 18 to 80 years, and all cases were confirmed by histology as NPC. Tumor staging was performed according to the 7th American Joint Committee on Cancer. The flow chart for this study is shown in Figure 1. A total of 394 patients were included in the study. Patients’ medical information was obtained from medical records. Data on death was obtained by follow‐up. The need for informed consent was waived due to the retrospective nature of the study.

### 2.2 Body composition measurement

Body weight and height were measured with a medical scale at the time of admission. BMI was calculated as weight (in kg)/height^2^ (in m). Body surface area (BSA) is calculated using the Mosteller formula: BSA (m^2^) = ([Height (cm) × Weight (kg)]/3600)^1/2^.

### 2.3 Image analysis

The European Working Group on Sarcopenia in Older People recommends computed tomography (CT) and magnetic resonance imaging as the gold standard for muscle mass evaluation.^10^ Abdominal CT is often used for diagnostic staging and follow‐up monitoring for NPC patients. Abdominal CT scans were respectively performed at baseline and after CRT (or at the end of treatment). The third lumbar vertebra was selected as the standard plane of positioning and a single slice image was acquired. Image analysis was performed by ImageJ (ImageJ software, https://imagej.nih.gov/ij). The skeletal muscle areas (SMA, in cm^2^) of all the muscle tissues (including the rectus abdominis, intra‐abdominal oblique muscle, external oblique muscle, transverse abdominis muscle, paraspinal muscle and psoas muscle) of the third lumbar vertebrae were sum up for each image. All CT images were analyzed by a trained observer (JM). The skeletal muscle index (SMI, in cm^2^/m^2^) was calculated as SMA divided by height square (in m^2^).

### 2.4 Definition of sarcopenia

In this retrospective study, SMI cut‐off values according to Zhuang et al^11^ were used to define sarcopenia (for male and female patients were 40.8 and 34.9 cm^2^/m^2^, respectively). Low skeletal muscle mass was defined as sarcopenia (actually not rigorous, because strictly speaking, the diagnosis and staging of sarcopenia should base on muscle mass, strength and performance).^10^

### 2.5 Survival

Survival time was defined as the time from the date of diagnosis to death or last follow‐up date. Follow‐up time was defined as the time from the beginning of radiotherapy to the onset of death, loss to follow‐up, or the end of follow‐up (1 December 2018). Data analysis was conducted in March 2019.

### 2.6 Statistical analysis

Continuous variables are described as mean ± SD, or median and interquartile range (IQR) based on the data characteristics. Classification variables or grade variables were described using frequency or composition ratio descriptions. A Wilcoxon signed‐rank sum test, Mann‐Whitney *U* test or corrected *t* test was used for skewed or variance data. Pearson's chi‐square test was used for comparisons between categorical variables or grade variables. The Kaplan‐Meier method was used to test the effect of each variable on survival. Log‐rank test is used to compare the survival curves of variables. After passing the proportional hazard (PH) assumption test, variables known to affect overall survival (OS) were included in the multivariate Cox PHs model, and the hazard ratios (HRs) and their 95% confidence intervals (CIs) were reported. C‐statistics were used to evaluate the discriminatory ability of the model, and the Hosmer‐Lemeshow goodness‐of‐fit test was used to evaluate the calibration ability of the predictive models. All statistical analyses were performed using IBM SPSS Statistics for Mac (Version 24.0; IBM Corp.) and graphs were constructed using GraphPad Prism® 8 (GraphPad Software, Inc). All *P*‐values were bilateral, and *P* ＜ .05 was considered statistically significant for all analyses.

The Cox univariate analysis included the main tumor prognostic indicators used in the current study: gender (male vs female), age (≤46 years vs >46 years), BMI grade (<24 kg/m^2^ vs ≥24 kg/m^2^), tumor staging (stage I‐II vs stage III‐IVB), pretreatment sarcopenia (yes vs no), posttreatment sarcopenia (yes vs no), and skeletal muscle loss (<15% vs ≥15%).

## 3 RESULTS

### 3.1 Patient characteristics

The average age of the participants was 46 (range, 18‐79) years‐old. A larger number of women were defined as sarcopenia (49.0%) at baseline than men (27.9%). All patients underwent radical intensity‐modulated radiotherapy with a median dose of 72.32 Gy. The patients with late‐T or late‐N stage receiving induction chemotherapy plus concurrent CRT (CCRT). Induction chemotherapy plus CCRT and CCRT accounted for 47.2% and 43.1% of all the patients, respectively. Patients' baseline characteristics are shown in Table 1.

**Table 1:** Baseline patient characteristics (N = 394)

Characteristics	No. (%)
Age, y	
<60	350 (88.8)
≥60	44 (11.2)
Gender	
Male	298 (75.6)
Female	96 (24.4)
BMI categories	
Underweight (<18.5 kg/m^2^)	34 (8.6)
Normal (18.5‐23.9 kg/m^2^)	238 (60.4)
Overweight (24.0‐27.9 kg/m^2^)	98 (24.9)
Obesity (≥28.0 kg/m^2^)	24 (6.1)
Education level	
High school graduate and below	320 (81.2)
Associate/bachelor's degree	74 (18.8)
Cancer stage (the 7th AJCC)	
I	4 (1.0)
II	66 (16.7)
III	117 (29.7)
IVA	133 (33.8)
IVB	74 (18.8)
T stage	
T1	25 (6.4)
T2	128 (32.5)
T3	90 (22.8)
T4	151 (38.3)
N stage	
N0	23 (5.8)
N1	167 (42.4)
N2	126 (32.0)
N3	78 (19.8)
Treatment regimen	
Radical radiotherapy ± TT	44 (11.2)
CCRT ± TT/AC	168 (42.6)
IC + CCRT ± TT/AC	177 (44.9)
IC + radical radiotherapy	5 (1.3)
SMI < reference value^a^	130 (33.0)

Abbreviations: AC, adjuvant chemotherapy; AJCC, American Joint Committee on Cancer; BMI, body mass index; CCRT, concurrent chemoradiotherapy; IC, induction chemotherapy.

a Sex‐specific cutoff values according to Zhuang et al.^11^

The incidence of sarcopenia at the baseline was 33.0% (130/394), and this value increased to 61.2% (241/394) after treatment. A paired chi‐square test (McNemar's test) showed that the difference in the proportion of sarcopenia before and after treatment was statistically significant (*P* < .001). There were significant differences in age, gender, BSA, BMI, SMA, SMI, hemoglobin, serum creatinine and urea acid between the two groups before or after treatment which is shown in Table 2.

**Table 2:** Pretreatment and posttreatment comparisons with two subgroups

Characteristics	Pretreatment sarcopenia			Posttreatment sarcopenia		
	Yes (n = 130)	No (n = 264)	*P*‐value	Yes (n = 241)	No (n = 153)	*P*‐value
Gender (n)			**＜.001**			**＜.001**
Male	83	215		163	135	
Female	47	49		78	18	
Age, y	49.4 ± 11.8	44.5 ± 10.8	**＜.001**	47.6 ± 12.0	43.6 ± 9.9	**＜.001** a
BSA, m^2^	1.605 ± 0.158	1.699 ± 0.167	**＜.001**	1.558 ± 0.149	1.667 ± 0.165	**＜.001**
L3‐SMA, cm^2^	92.9 ± 17.5	127.7 ± 23.2	**＜.001** a	91.7 (76.7‐104.2)	124.4 (114.2‐134.8)	**＜.001**
L3‐SMI, cm^2^/m^2^	34.5 (31.2‐37.8)	46.1 (42.5‐51.1)	**＜.001**	33.6 (30.0‐37.2)	44.9 (42.1‐48.6)	**＜.001**
BMI, kg/m^2^	21.0 ± 2.9)	23.4 ± 3.1	**＜.001**	19.5 (18.0‐21.3)	22.1 (20.4‐24.3)	**＜.001**
BMI categories (n)			**＜.001**			**＜.001**
<24	111	164		226	113	
≥24	19	100		15	40	
Cancer stage (n)			.153			.065
I‐II	18	52		36	34	
III‐IVB	112	212		205	119	
T stage (n)			**.037**			.069
T1‐2	41	112		85	68	
T3‐4	89	152		156	85	
N stage (n)			.882			.280
N0‐1	62	128		111	79	
N2‐3	68	136		130	74	
HGB, g/L	136.0 (122.8‐147.0)	143.0 (131.3‐152.0)	**＜.001**	103.8 ± 19.3	110.7 ± 17.8	**＜.001**
ALB, g/L	40.5 ± 3.3	40.9 ± 3.4	.217	37.0 (34.2‐39.2)	37.8 (35.7‐39.5)	**.042**
PA, mg/L	252.5 (206.8‐301.5)	271.0 (232.3‐307.8)	.052	187.4 ± 62.5	206.0 ± 64.3	**.005**
SCR, μmol/L	70.6 ± 15.4	76.3 ± 14.8	**＜.001**	70.0 (59.0‐84.0)	78.0 (68.5‐91.0)	**＜.001**
Urea, mmol/L	4.65 (3.85‐5.63)	4.62 (3.72‐5.32)	.545	3.50 (2.61‐4.88)	4.00 (2.95‐5.26)	**.013**
UA, μmol/L	312.1 ± 81.2	354.1 ± 92.4	**＜.001**	266.0 (207.5‐324.0)	281.0 (222.0‐352.5)	**.048**
Status(n)			.622			.473
Alive	113	234		210	137	
Dead	17	30		31	16	

Note

The values of the bold label are statistically significant.

Abbreviations: ALB, albumin; BMI, body mass index; BSA, body surface area; HGB, hemoglobin; SCR, serum creatinine; SMA, skeletal muscle area; SMI, skeletal muscle index; PA, prealbumin; UA, uric acid.

a For adjusted *t* test.

### 3.2 Skeletal muscle loss

Sarcopenia can occur in more than 91% of people who are not underweight (normal, overweight, or obese). There was no significant difference in the effect of the SMA between the different treatment regimens (*P* = .270). There was no significant difference in the effect of different chemotherapy cycles on the skeletal muscle loss rate (*P* = .422), but the explanatory power of this conclusion is weakened due to the presence of radiotherapy.

**Figure 2:**
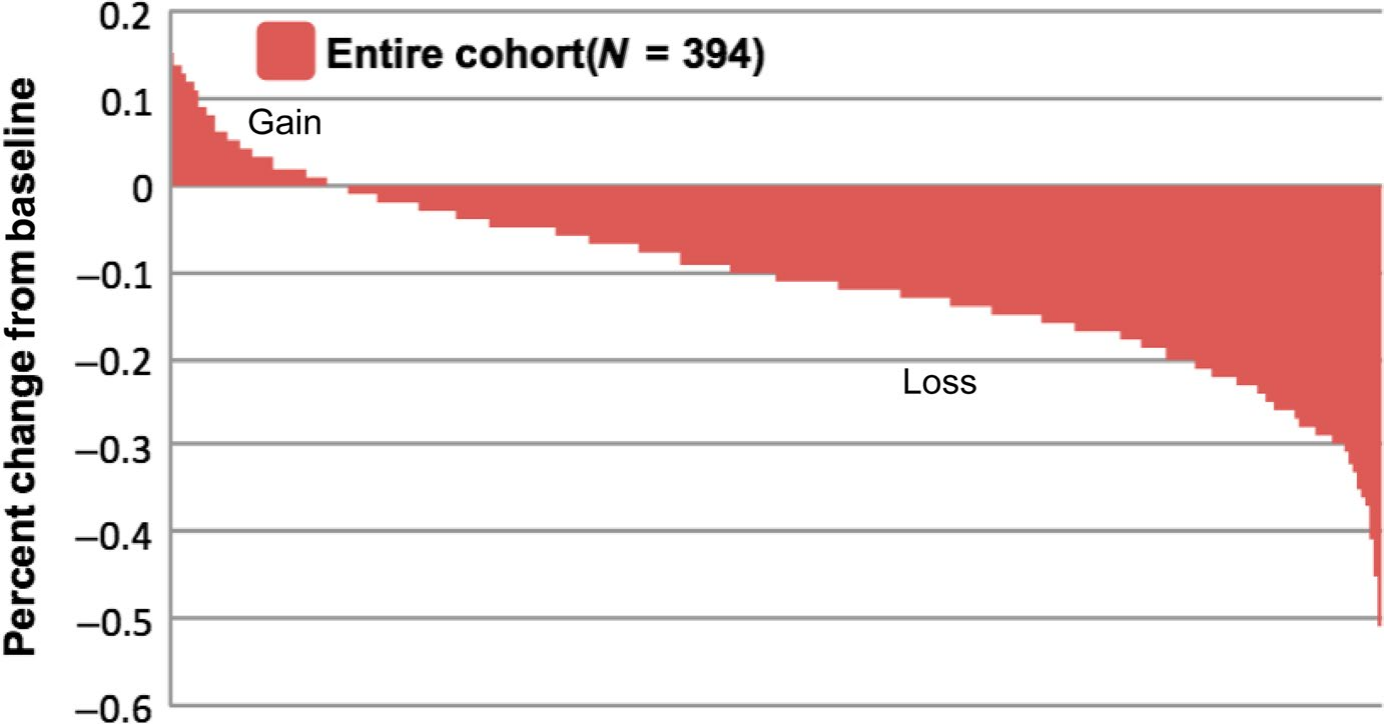
The waterfall plot shows the distribution of muscle change rate after chemoradiotherapy in all patients compared to baseline

Due to individual differences in the relative muscle change, we used the receiver operating characteristic curve based on the post‐treatment skeletal muscle reduction rate to obtain the most approximate value of the Youden index (15.5%), then removed the last decimal place for classification. According to the cut‐off value, the degree of skeletal muscle loss was divided into mild‐to‐moderate muscle loss (MML, <15%) and severe muscle loss (SML, ≥15%). A total of 119 patients (30.2%) developed SML after CRT. The vast majority of patients experienced varying degrees of muscle loss after CRT. Figure 2 shows the rate of muscle change in all patients. The upper and lower parts of the zero point represent muscle weight gain and loss, respectively. As can be seen in the Figure 2, the rate of muscle loss in most patients ranges from 0.05 to 0.20.

**Figure 3:**
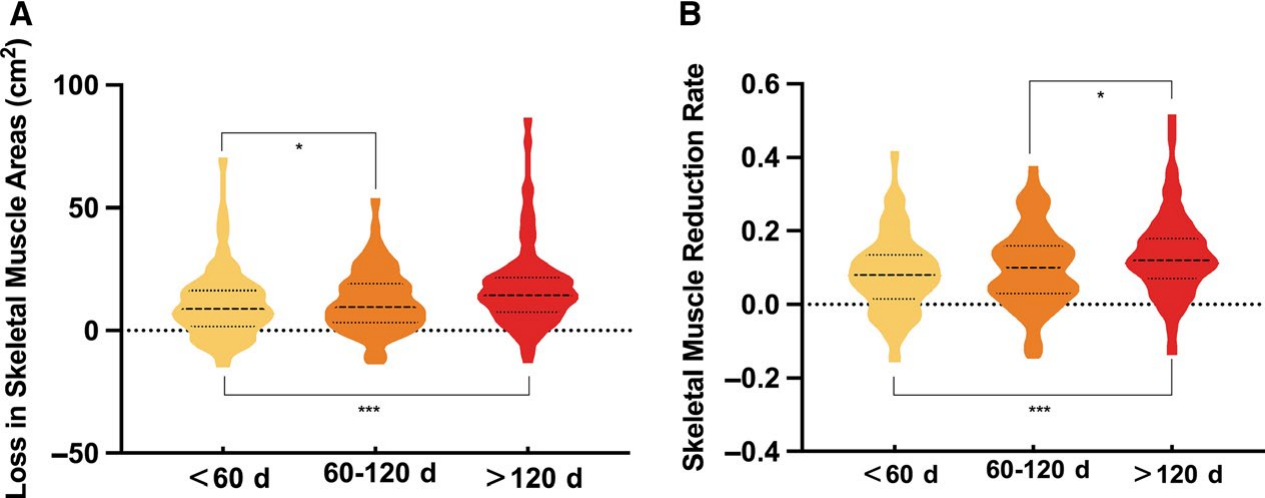
(A, B) respectively show the difference of skeletal muscle reduction area and rate in different periods of second computed tomography scan. The bold lines represent the median, while the upper and lower thin lines represent upper quartile (Q3) and lower quartile (Q1), respectively. ^*^ *P* < .05, ^***^ *P* < .001

According to the time interval between two CT scans (median: 110 days; IQR: 59‐106 days; range: 41‐1083 days), the whole queue was divided into three groups: <60 days group, 60‐120 days group, >120 days group. The degree of muscle tissue loss, as observed in the second CT scan time (divided into <60 days, 60‐120 days, and >120 days), is shown in Figure 3. By Kruskal‐Wallis test, *H* = 19.084, *P* < .001, the difference in muscle loss at three different review points was considered to be different. After a pairwise comparison, the difference in muscle loss between the <60‐day group and the >120‐day group was statistically significant (adjusted *P* < .001), and the difference in muscle size from the 60‐120 day group was also statistically significant (adjusted *P* = .017); The difference between the 60‐120 day group and the >120 day group was not statistically significant (adjusted *P* = .762). Therefore, we believe that the point of review has an effect on the amount of skeletal muscle loss. Regardless of muscle loss quantity or reduction rate, there was a statistically significant difference (*P* < .001) in the longitudinal changes between the muscles after 4 months of treatment (>120 days group) and the first 2 months (<60 days group).

Cox univariate analysis was used to assess OS‐related skeletal muscle loss, regardless of continuous variables (HR 1.000, 95% CI 0.999‐1.001, *P* = .753) or grade variables (*P* = .257), there was no statistically significant effect of CT interval on survival. According to previous analysis, we believe that CT interval has an effect on the degree of skeletal muscle loss. In addition, by K‐M method, the chi‐square value of three different CT intervals was 3.605, *P* = .165, indicating that there was no statistical difference in the effect on survival. Therefore, it was not included in the covariate analysis.

### 3.3 Changes in SMA and SMI

Patients with sarcopenia increased from 33.0% at baseline to 61.2% after treatment. The skeletal muscle area of the entire cohort decreased by an average of 13.1 cm^2^ (95% CI 10.1‐13.4; *P* < .001) and the SMI decreased by 4.7 cm^2^/m^2^ (95% CI 4.1‐5.3; *P* < .001). After CRT, SMA and SMI were significantly reduced in male and female patients (Table 3).

**Table 3:** Changes in muscle area and L3‐SMI after CRT (N = 394)

Variables	First CT scan		Second CT scan		Decrease		
	Mean	SD	Mean	SD	Mean	95% CI	*P*‐value
Muscle area, cm^2^	116.2	27.0	103.1	24.5	13.1	10.1‐13.4	<.001^a^
Male	126.6	21.5	112.6	19.3	14.0	12.4‐15.6	<.001^a^
Female	84.1	13.7	73.7	12.6	10.4	8.2‐12.5	<.001^a^
L3‐SMI, cm^2^/m^2^	42.8	8.4	38.1	8.0	4.7	4.1‐5.3	<.001^a^
Male	45.4	7.5	40.4	6.8	5.0	4.4‐5.8	<.001
Female	34.7	5.5	30.9	7.0	3.8	2.4‐5.2	<.001^a^

Abbreviations: CT, computed tomography; SD, standard deviation; SMI, skeletal muscle index.

a Mann‐Whitney *U* test

### 3.4 Overall survival

The median follow‐up time was 22.7 (range 2.5‐46.4) months, the overall follow‐up rate was 96.2%. The 3‐year OS rate was 81.2%, and the entire cohort survival rate was 88.1%. According to SMI, there was no significant difference in the 3‐year OS between the sarcopenia and non‐sarcopenia groups (pretreatment: 82.9% vs 80.3%, Log‐rank *χ* ^2^ = 0.115, *P* = .735; posttreatment: 81.0% vs 81.8%, Log‐rank *χ* ^2^ = 0.835, *P* = .361).

**Figure 4:**
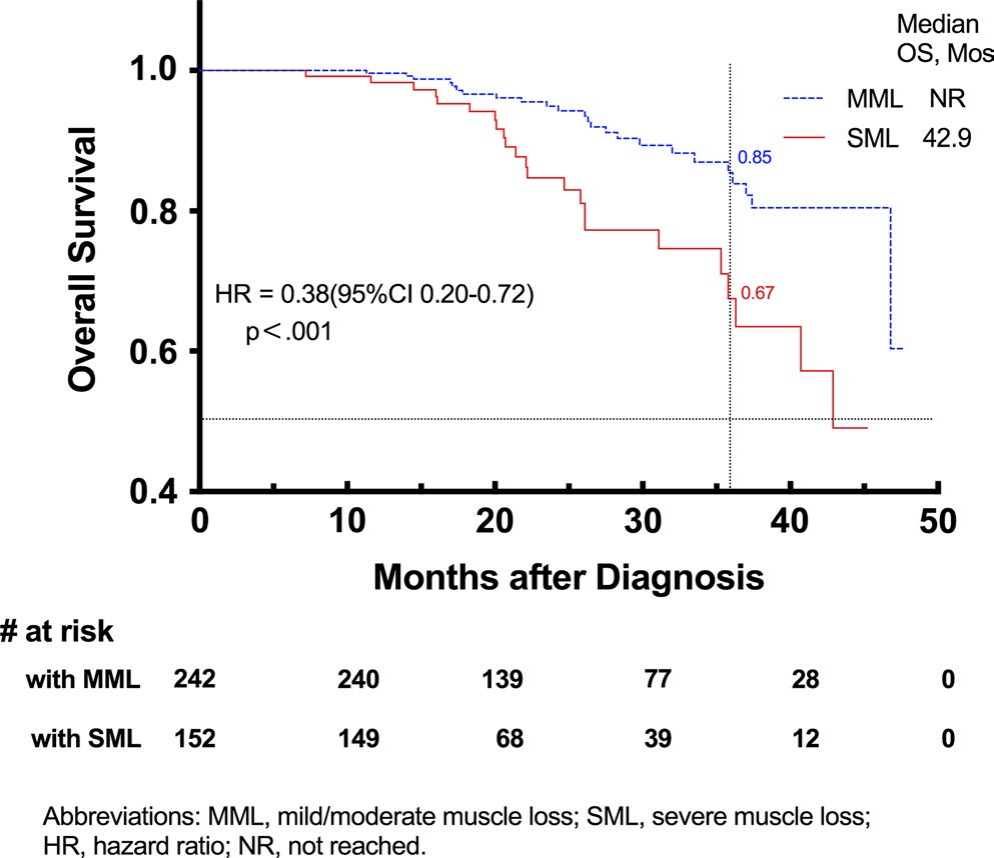
Kaplan‐Meier curve demonstrating overall survival according to skeletal muscle change groups for mild‐to‐moderate muscle loss (MML) and severe muscle loss (SML) patients (log‐rank test, *χ* ^2^ = 12.07, *P* = .0005)

**Figure 5:**
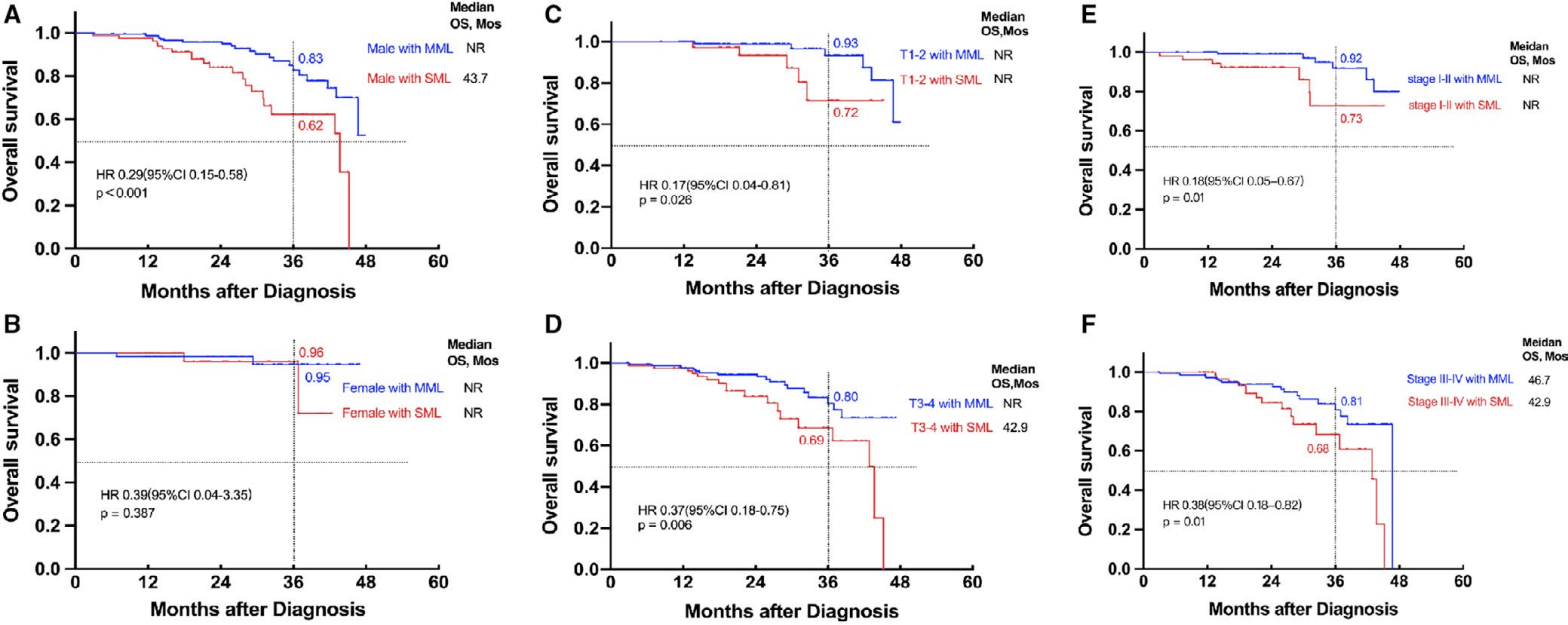
Stratified analysis for gender, T stage and cancer stage. As is shown in the figure, those patients with severe muscle loss (SML) during treatment have lower overall survival (OS) than the ones with mild‐to‐moderate muscle loss (MML) except for female. A and B respectively describe men and women; C and D respectively describe T1‐2 and T3‐4; E and F describe stages I‐II and III‐IV, respectively

The median survival of the patients with severe skeletal muscle loss was 42.9 months (95% CI 42.3‐45.1 months). The Kaplan‐Meier survival curves of the two groups are shown in Figure 4. Patients with SML during CRT had significantly lower OS rates than those with MML. The 3‐year OS of these two groups was 67% and 85%, respectively. In the stratified analysis, there was no significant difference in OS between women with different degrees of skeletal muscle loss. However, for men, different T‐stages, and different cancer stages, patients with SML had a shorter OS than MML patients (Figure 5).

By univariate analysis (chi‐square test), the educational level had no significant effect on survival status (Pearson *χ* ^2^ = 2.320, *P* = .128); in addition, by Cox univariate analysis, there was no significant difference in the survival of different educational levels (*P* = .336) (data is not shown in the manuscript). Therefore, the education level is not included in the multi‐factor analysis. On the other hand, by univariate analysis, age has an effect on survival (HR 1.040, 95% CI 1.013‐1.067, *P* = .004), but the age HR value is close to 1 (meaning the risk is equal to the average) Therefore, in the Cox univariate analysis, we divide the ages into two groups of ≤46 years and >46 years by the average, and the resulting HR value is 1.70 (95% CI 0.95‐3.05, *P* = .075). Therefore, it is not included in the multivariate model.

Data on the Cox univariate analysis and prediction models for each parameter are shown in Table 4. Under the premise that the C statistics and their 95% CIs are similar, Model 3 was considered as having greater calibration ability than the former two, for the sake of streamlined model considerations. Therefore, this study finally adopted Model 3 for the prediction of survival prognoses in NPC patients.

**Table 4:** Univariate and multivariate cox regression analysis

Variables	No. of patients	No. of events	Univariate Cox					Multivariate model 1					Multivariate model 2					Multivariate model 3				
			B	SE	HR	95% CI	*P*	B	SE	HR	95% CI	*P*	B	SE	HR	95% CI	*P*	B	SE	HR	95% CI	*P*
Gender																						
Female	96	4	Ref.																			
Male	298	43	1.28	0.52	3.60	1.29‐10.04	.014	1.08	0.53	2.96	1.05‐8.34	.041						1.53	0.55	4.60	1.58‐13.44	.005
Age, y																						
≤46			Ref.																			
>46			0.53	0.30	1.70	0.95‐3.05	.075															
Cancer stage																						
I‐II	187	13	Ref.																			
III‐IVB	207	34	0.90	0.33	2.45	1.29‐4.66	.006	0.89	0.34	2.43	1.26‐4.68	.008	0.93	0.34	2.52	1.31‐4.86	.006	0.98	0.35	2.66	1.34‐5.29	.005
BMI, kg/m^2^																						
<24	339	36	Ref.																			
≥24	55	11	1.01	0.35	2.76	1.40‐5.44	.003	0.98	0.36	2.66	1.33‐5.34	.006	1.15	0.35	3.16	1.59‐6.92	.001					
Muscle loss, %																						
<15	275	24	Ref.																			
≥15	119	13	1.05	0.30	2.84	1.59‐5.44	<.001	0.96	0.30	2.62	1.46‐4.70	.001	1.02	0.33	2.79	1.47‐5.28	.002	1.02	0.33	2.79	1.47‐5.28	.002
Pretreatment																						
Non‐sarcopenia	264	30	Ref.																			
Sarcopenia	130	17	0.10	0.30	1.11	0.61‐2.01	.735															
Posttreatment																						
Non‐sarcopenia	153	16	Ref.																			
Sarcopenia	241	31	0.28	0.31	1.33	0.72‐2.44	.363															
C‐Statistic (95% CI)								0.730 (0.657‐0.802)					0.684 (0.607‐0.761)						0.719 (0.646‐0.793)			
χ^2^ (*P*‐value)								0.660‐0.779 (0.901)					1.342 (0.854)						0.954 (0.966)			

Note

Univariate and multivariate Cox regressions. Generally speaking, AUC <0.60 is considered to have poor discriminability, 0.60‐0.75 is considered to have some discriminating ability, and >0.75 is considered to have good discriminating ability.

## 4 DISCUSSION

Our study shows that for NPC patients undergoing radical CRT, severe skeletal muscle loss during treatment may be an important predictor of OS. The patients with severe skeletal muscle loss had an increased all‐cause mortality compared to those with mild‐to‐moderate skeletal muscle loss (adjusted HR 2.79, 95% CI 1.47‐5.28, *P* = .002), indicating a certain mechanism between skeletal muscle change and survival. The degree of skeletal muscle loss has better prognostic value than another risk factors, such as age, gender, cancer stage, BMI, BSA, and weight loss.

Skeletal muscle wasting begins early in life,^12^ especially in people with a sedentary lifestyle.^13^, ^14^ Lean body tissue (mainly skeletal muscle) is the largest protein storage site in the body. Whether during malignant tumor development^15^ or cancer treatment,^5^, ^6^, ^9^, ^16^ a certain amount of skeletal muscle loss could occur. The degree of muscle loss, as confirmed in a variety of solid tumor studies, is severely underestimated by clinical oncologists. Skeletal muscle loss that occurs during cancer presence and its treatment may have a different mechanism of action than age‐related skeletal muscle loss. Numerous animal models and clinical studies have shown that sarcopenia and cachexia can be mediated by mechanisms such as systemic inflammation,^7^ oxidative stress,^17^ autophagy,^18^ and adipose tissue metabolic disorder.^19^ In current study, the difference in the skeletal muscle reduction rate between the different treatment regimens was not statistically significant (Pearson *χ* ^2^ = 4.006, *P* = .549), and the effect of different chemotherapy cycles on SMA reduction was also not statistically significant (Kruskal‐Wallis test, *χ* ^2^ = 2.288, *P* = .319), suggesting that radiotherapy may play a dominant role in skeletal muscle loss. The incidence of sarcopenia before and after treatment in this cohort (33% and 61.2%, respectively) was similar to that previously published study by the US Anderson Cancer Center (35.3% and 65.8%, respectively).^9^ It is suggested that in the death‐related multifactor model, skeletal muscle loss after radiotherapy is more substantial than weight loss (Bayesian information criterion difference, 7.9), further supporting our findings. The Kaplan‐Meier method showed that patients with sarcopenia who were overweight/obese (BMI ≥ 24 kg/m^2^) did not show worse survival than those with non‐sarcopenia (log‐rank *χ* ^2^ = 0.772, *P* = .394). This is in contrast to the findings of previously published studies^20^, ^21^; this may be related to the inconsistencies in the sample size and the cut‐off point of sarcopenic obesity.

Although severe skeletal muscle loss during treatment significantly increased the risk of death in patients with NPC, there was no association between sarcopenia and OS. This result was supported by Susanne et al^6^ who divided the muscle changes into three groups based on relative muscle changes every 3 months, and then merged the two subgroups with no significant differences, and finally determined the critical value of 9%. In another study on patients with radical cystectomy for bladder cancer,^22^ sarcopenia increased the risk of postoperative complications in women, but it was not associated with OS. Body composition largely differs depending on age, gender, and ethnicity,^23^ which may have a significant effect on the degree of skeletal muscle loss.

In addition, we noticed that of 17 patients who did not meet the diagnostic criteria (non‐sarcopenia) but with severe skeletal muscle loss during treatment, 4 died (data not shown), suggesting that this group still have a high risk of death. Over time, the effects of severe skeletal muscle loss on long‐term survival may be more pronounced. Therefore, we believe that compared with the term of sarcopenia, the dynamic changes of skeletal muscle are deserved more attention to clinical oncologists in predicting the survival of patients with cancer treatment or other clinical outcomes.

In this predictive model, the only variable that can be adjusted is skeletal muscle mass. An important finding of our study, which has clinical applicability, is that the performance of CT and assessment of longitudinal skeletal muscle changes 4 months after treatment may sensitively screen those at risk of muscle nutrition. Some necessary interventions, such as exercise^24^ and nutritional intervention,^25^ drug therapy,^26^, ^27^ or other clinical interventions may be of great benefit to cancer survivors. However, whether these interventions can improve long‐term survival remains to be supported by the conclusions of future large‐scale prospective studies.

### 4.1 Limitations

This study has some limitations. First, due to insufficient follow‐up time, the conviction of the current research conclusions has been somewhat affected. Second, owing to its retrospective design, it was impossible to obtain data on some important diagnostic factors of sarcopenia such as grip strength, walking speed, and patient self‐report.^10^ In addition, not all patients underwent CT scan as a follow‐up examination, limiting the study's sample size. Besides, the second CT scan time was inconsistent, resulting in the inconsistency of the skeletal muscle loss between patients. In this study, we did not quantify skeletal muscle density (SMD). Therefore, it is failed to comprehensively evaluate skeletal muscle from the two dimensions including SMI (quantity) and SMD (quality). These shortcomings cause certain bias and need to be strictly designed in the future.

## 5 CONCLUSIONS

In this retrospective cohort study, we conducted a preliminary exploration of the critical value of longitudinal skeletal muscle changes in Chinese NPC patients. In general, varying degrees of skeletal muscle loss are commonly observed in NPC patients undergoing radical CRT. Patients with severe muscle loss were associated with a reduction in OS. Female patients seem to be more susceptible to sarcopenia, but different degree of muscle loss has no significant difference in OS. In men, in different T stages and tumor stages, the OS of patients with severe muscle loss was significantly shortened. Clinical oncologists need to fully consider that patients with the same BSA or the same BMI may have skeletal muscle mass differences, resulting in distinct toxicities or survival. In addition, we believe that the 4 months after treatment is appropriate timepoint for the reassessment of the L3‐SMA and SMI, and screening of high‐risk populations. Despite some limitations, our findings still increase the likelihood of the incorporation of skeletal muscle tissue changes into the prognostic risk model for NPC. Moreover, terms such as skeletal muscle loss and sarcopenia should be further distinguished and standardized in order to avoid mixing together. Future research should focus on the prediction of the cut‐off value of skeletal muscle loss in terms of survival and other clinical outcomes (eg, quality of life, metastasis, recurrence, etc) to better guide individualized clinical practice.

## CONFLICT OF INTEREST

None declared.

## AUTHOR CONTRIBUTIONS

Conception and design of study: Xiao Huang, Xiao‐Dong Zhu; imaging data analysis: Jie Ma; acquisition, analysis, or interpretation of data: all authors; drafting of the manuscript: Xiao Huang; critical revision of the manuscript for important intellectual content: all authors; statistical analysis: Xiao Huang.
